# Congenital Anomalies in Children Exposed to Antithyroid Drugs *In-Utero*: A Meta-Analysis of Cohort Studies

**DOI:** 10.1371/journal.pone.0126610

**Published:** 2015-05-14

**Authors:** Huixia Li, Jianfei Zheng, Jiayou Luo, Rong Zeng, Na Feng, Na Zhu, Qi Feng

**Affiliations:** 1 Department of Maternal and Children Health, School of Public Health, Central South University, Changsha, Hunan Province, China; 2 Department of Emergency and Intensive Care Medicine, The second Xiangya Hospital, Central South University, Changsha, Hunan Province, China; 3 Department of Pharmacy, Xiangya Hospital, Central South University, Changsha Hunan Province, China; Kuopio University Hospital, FINLAND

## Abstract

**Background:**

Hyperthyroidism affects about 0.2%-2.7% of all pregnancies, and is commonly managed with antithyroid drugs (ATDs). However, previous studies about the effects of ATDs on congenital anomalies are controversial. Therefore, the present meta-analysis was performed to explore the risk of congenital anomalies in children exposed to ATDs in-utero.

**Methods:**

Embase, Pubmed, Web of Knowledge, and BIOSIS Citation Index were searched to find out studies about congenital anomalies in children exposed to ATDs in-utero reported up to May 2014. The references cited by the retrieved articles were also searched. The relative risks (RRs) and confidence intervals (CIs) for the individual studies were pooled by fixed effects models, and heterogeneity was analyzed by chi-square and *I* ^2^ tests.

**Results:**

Eight studies met the inclusion criteria. Exposure to propylthiouracil (PTU), methimazole/carbimazole (MMI/CMZ), and PTU & MMI/CMZ was investigated in 7, 7 and 2 studies, respectively. The pooled RR was 1.20 (95%CI: 1.02-1.42), 1.64 (95%CI: 1.39-1.92), and 1.83 (95%CI: 1.30-2.56) for congenital anomalies after exposure to PTU, MMI/CMZ, and PTU & MMI/CMZ, respectively.

**Conclusions:**

The meta-analysis suggests that exposure to ATDs in-utero increases the risk of congenital anomalies. The use of ATDs in pregnancy should be limited when possible. Further research is needed to delineate the exact teratogenic risk for particular congenital anomaly.

## Introduction

Clinical hyperthyroidism which is a common endocrinopathy in pregnancy affects about 0.2%- 2.7% of all pregnancies in the world [1–4]. The most common type of hyperthyroidism is Graves’ disease. Poorly-controlled hyperthyroidism during pregnancy is associated with recurrent miscarriage, preeclampsia, intrauterine growth restriction, preterm delivery, low birth weight, and haemorrhage in the postpartum period [5,6]. Therefore, hyperthyroidism in pregnant women should be treated appropriately. Antithyroid drugs (ATDs) including propylthiouracil (PTU), methimazole (MMI), and carbimazole (CMZ, prodrug to MMI) are the first line treatment of hyperthyroidism in pregnant women [7,8]. These drugs have equal effects in the treatment of prenatal hyperthroidism. However, they are known to cross the human placenta [9] and thus may affect the fetus.

Many observational studies have assessed the effects of ATDs on the occurrence of congenital anomalies, but the results are conflicting. Several case reports and some epidemiologic studies suggest that exposure to MMI/CMZ in the first trimester is associated with an increased risk of congenital anomalies, including abdominal wall defect[10], aplasia cutis congenita[10–14], choanal atresia[10,14–16], tracheo- oesophageal fistula[14], and omphalocele[15]. A recent case-control study shows that prenatal exposure to PTU is associated with situs inversus dextrocardia, isolated unilateral kidney, and cardiac outflow tract defects [15]. The largest retrospective cohort study to date, which was conducted in Denmark, indicates that exposure to PTU, MMI/CMZ or both in the first trimester is associated with an increased risk of congenital anomalies[17]. However, several small- to medium- scale cohort studies do not find the association between prenatal exposure to ATDs and increased risk of congenital anomalies[2,5,18,19].

The objective of the present meta-analysis is to investigate the effects of ATDs on congenital anomalies, to find out whether exposure to ATDs in-utero is associated with increased risk of congenital anomalies, and thus to provide an overall assessment of ATDs safety and give a guideline for doctors when they prescribe these drugs to hyperthyroid pregnant women.

## Materials and Methods

### Data sources

Embase, Pubmed, Web of Knowledge, and BIOSIS Citation Index were searched to find out studies about congenital anomalies in children exposed to ATDs in-utero. The studies were reported between 1950 to May 2014. The keywords used to search for studies about the exposure to ATDs were “antithyroid agents”, “antithyroid drugs”, “propylthiouracil”, “methylthiouracil”, “methimazole” and “carbimazole”; the keywords used to search for outcomes were “pregnancy outcomes”, “birth defects”, “congenital malformations”, “congenital abnormalities”, “abnormalities-drug induced”, and “congenital anomalies”. The references cited by the retrieved articles were also searched manually to find out additional articles.

### Study selection

In this meta-analysis, the selected studies needed to meet the following criteria: 1) Prospective or retrospective cohort study about children whose mothers were treated for hyperthyroidism during pregnancy; 2) Exposure to ATDs in-utero (6 months before pregnancy to the end of pregnancy); 3) Interested outcomes were major and/or minor congenital anomalies, excluding chromosomal anomalies and other anomalies of known aetiology; 4) Incidence of congenital anomalies reported; 5) Studies written in any language with an English abstract.

Any study meeting a definite exclusion criterion was excluded from the meta-analysis: 1) Not about children whose mothers were treated for hyperthyroidism during pregnancy; 2) Animal study; 3) Case-control study; 4)Review, letter to editor, news, editorial, commentary, or case report; 5) No outcome of interest reported; 6) No control group or an inappropriate control group.

Study eligibility was determined by two reviewers, who screened all titles, abstracts of the retrieved citations and full papers when necessary. All inclusion criteria and no exclusion criterion should be met for studies to get into the final meta-analysis.

### Data extraction

Based on the preliminary screening, those studies meeting the inclusion criteria but not meeting any exclusion criterion were included in the meta-analysis. Two reviewers independently extracted information from each eligible study, including first author, publication year, study location, study design, treatment characteristics, control cohort, outcome, numbers in the exposed cohort and the control cohort, events of congenital anomalies in both cohorts, and methodological quality. The methodological quality of cohort studies was assessed on basis of Newcastle-Ottawa Scale (NOS). NOS consisted of three domains and 8 items: 4 items for selection, 1 item for comparability, and 3 items for outcome [20]. Any disagreement between the two reviewers was solved on basis of the assessment of a third reviewer.

### Statistical analysis

The pooled relative risks (RRs) and 95% confidence intervals (CIs) were calculated using the Mantel-Haenszel fixed model or the random effects model, depending on the heterogeneity of the included studies. Heterogeneity was assessed by both Cochran chi-square test, with *P* ≤ 0.10 as a significance level, and *I* ^2^ test, with <25%, 25–50%, and >50% indicating low, moderate and high heterogeneity respectively [21,22]. Publication bias was examined visually from funnel plots, and formally quantified by the fail-safe N (N*fs*). The fail-safe N computes the numbers of missing studies that would bring the *P*-value to larger than 0.05. Sensitivity analysis was performed to evaluate the stability and reliability of the results. Significance was set at *P* < 0.05. All statistical analyses were performed using Cochrane’s Review Manager 5.0 (Oxford, England: Cochrane Collaboration).

## Results

### Study selection and study characteristics

**Fig 1:**
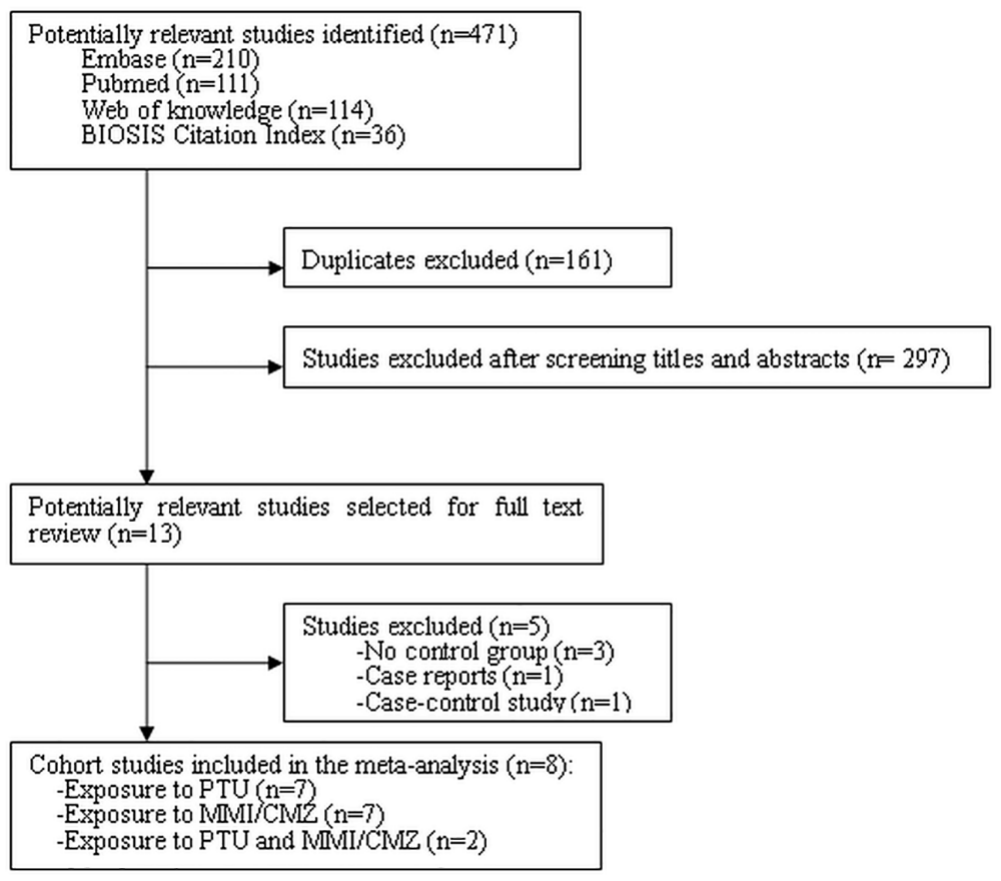
Flow chart of the study selection process.

Based on our search strategy, 471 articles were initially identified, with 210 from Embase, 111 from Pubmed, 114 from Web of Knowledge, and 36 from BIOSIS Citation Index. After removal of duplicates and the initial screening, 13 potentially relevant articles were included for detailed review. Five articles[10, 15,23–25] did not meet the inclusion criteria, and thus, 8 articles [2,3,5,17–19,26,27] were included in the meta-analysis. Fig 1 shows the study selection process.

Table 1 presents the 8 articles and their characteristics. The 8 full papers addressing our research issue were published between 1994 and 2013. Seven studies separately reported PTU exposure cohorts[2,3,5,17,18,26,27], 7 studies reported MMI/CMZ exposure cohorts[2,3,17–19,26,27], and 2 studies reported PTU & MMI/CMZ exposure cohorts [2,17].

**Table 1:** Characteristics of included studies.

Study	Type of study	Exposed cohort		Unexposed cohort		Duration of exposure	Publication year	Study location
		Subgroup	Number of anomalies/total	Subgroup	Number of anomalies/total			
Andersen et al	Retrospective cohort study	PTU, MMI/CMZ, PTU and MMI/CMZ	45/564, 100/1097, 16/159	No ATD and without hyperthyroidism	45982/811730	6 months before pregnancy to the end of the 10^th^ gestational week	2013	Denmark
Korelitz et al	Retrospective cohort study	PTU, MMI, PTU and MMI	66/915, 6/108, 14/126	No ATD and without thyrotoxicosis	37351/634858	6 months before pregnancy to the end of pregnancy	2013	Unite State
Yoshihara et al	Retrospective cohort study	PTU, MMI	26/1578, 50/1426	No ATD but Graves’ disease	40/2065	During the first trimester of pregnancy	2012	Japan
Chen et al	Retrospective cohort study	PTU, MMI	5/630, 0/73	No ATD and without hyperthyroidism	92/14150	During pregnancy	2011	Taiwan
Rosenfeld et al	Retrospective cohort study	PTU	1/80	No ATD but exposed to nonteratogenic drugs	34/1066	Between the 4^th^ and 13^th^ gestational week	2009	Israel
Lian et al	Retrospective cohort study	PTU, MMI	1/28, 5/12	No ATD and with hyperthyroidism	1/61	During the first trimester of pregnancy	2005	China
Gianantonio et al	Prospective cohort study	MMI/CMZ	8/241	No ATD but exposed to nonteratogenic drugs	23/1089	During pregnancy	2001	Europe
Wing et al	Retrospective cohort study	PTU, MMI	3/99, 1/36	No ATD with/without hyperthyroidism	1/43	During pregnancy	1994	Unite State

Note: One study reported the outcomes with all congenital anomalies coded in ICD-9 (Korelitz et al.), one study reported the outcomes with congenital anomalies but without a clear definition (Lian et al.), and other 6 studies reported their outcomes with major congenital anomalies.

### Assessment of methodological quality

The overall score of methodological quality was 9 based on NOS for cohort studies, with four stars for selection, two stars for comparability, and three stars for outcome [20]. Table 2 shows the quality scores of the included studies.

**Table 2:** Assessment of methodological quality by NOS.

Study	Selection				Comparability	Outcome			Total
	Exposed cohort representativeness	Non exposed cohort Selection	Ascertainment of exposure	Outcome not present at start of study	Comparability of cohorts	Assessment of outcome	Follow-up long enough	Adequacy of follow up	
Andersen et al	▲	▲	▲	▲	▲▲	▲	▲	▲	9
Korelitz et al	▲	▲	▲	▲	▲	▲	▲	▲	8
Yoshihara et al	▲	▲	▲	▲	▲	▲	▲	▲	8
Chen et al	▲	▲	▲	▲	▲	▲	▲	▲	8
Rosenfeld et al	▲	▲	▲	▲	▲	▲	▲		7
Lian et al		▲	▲	▲		▲	▲	▲	6
Gianantonio et al	▲	▲		▲	▲		▲	▲	6
Wing et al	▲	▲	▲	▲	▲	▲	▲	▲	8

NOS: Newcastle-Ottawa Scale

▲: One score in the item

Two studies[19,26] contained mild cohort selection bias because the exposed cohort in one study was not well representative (hyperthyroid pregnant women exposed to ATDs in a hospital were selected as the exposed cohort) or because the exposure ascertainment was written self-report without secure record or structured interview. Five studies[2,3,5,18,19] had moderate comparability, because they did not fully adjust some confounding factors, including thyroid status of mothers, and the type, severity and duration of maternal hyperthyroidism. One study[26] had inferior comparability, and even did not report the maternal age in the exposed cohort or the unexposed cohort. Two studies [5,19] contained mild outcome bias, because the assessment of outcome was based on self-report of the participants in one study, and the adequacy of follow-up was not stated in the other study.

### Overall effect of congenital anomalies

**Fig 2:**
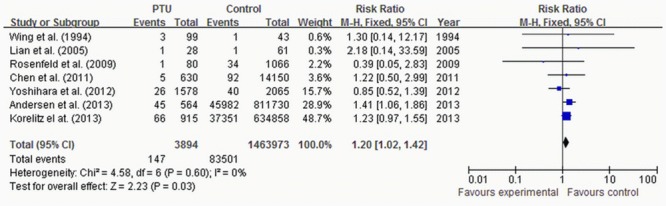
Risk of congenital anomalies for children exposed to PTU.

**Fig 3:**
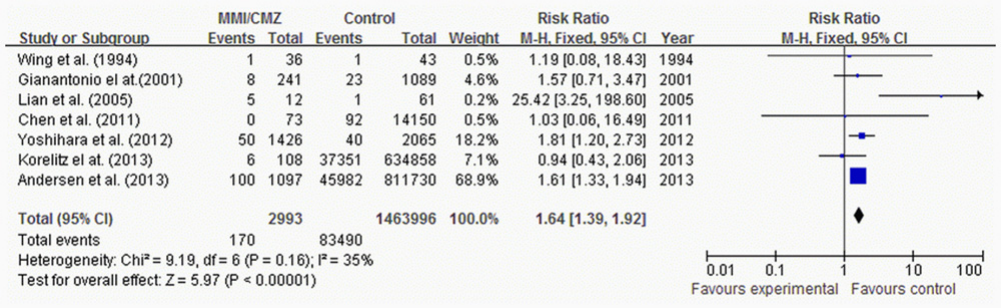
Risk of congenital anomalies for children exposed to MMI/CMZ.

**Fig 4:**
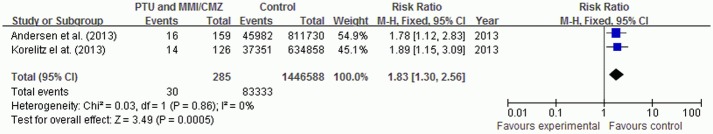
Risk of congenital anomalies for children exposed to PTU& MMI/CMZ.

Eight studies reported congenital anomalies. There were totally 3894, 2993, and 285 pregnant women exposed to PTU, MMI/CMZ and PTU & MMI/CMZ, respectively, corresponding to 1463973, 1463996 and 1446588 non-exposed pregnant women, respectively. The exposure typically started from 6 months before pregnancy to the end of pregnancy. The pooled RRs for congenital anomalies in exposure to different ATDs in-utero are shown in Figs 2 to 4.

The pooled RR for congenital anomalies was 1.20 (95%CI 1.02–1.42) in 7 studies reporting exposure to PTU (Fig 2), 1.64 (95%CI 1.39–1.92) in 7 studies reporting exposure to MMI/CMZ (Fig 3); and 1.83 (95%CI 1.30–2.56) in 2 studies reporting exposure to PTU & MMI/CMZ (Fig 4). The chi-square and *I* ^2^ tests for heterogeneity (*P* = 0.16~0.86, *I* ^2^ = 0~35%) indicated that the studies were homogeneous and could be combined, and thus the fixed effect models were applied to pool the RRs for meta-analysis of studies. All of the pooled RRs were significant, which indicated that exposure to PTU or/and MMI/CMZ in-utero would enhance the risk of congenital anomalies compared with the case of “without exposure to ATDs in-utero”.

### Publication bias

**Fig 5:**
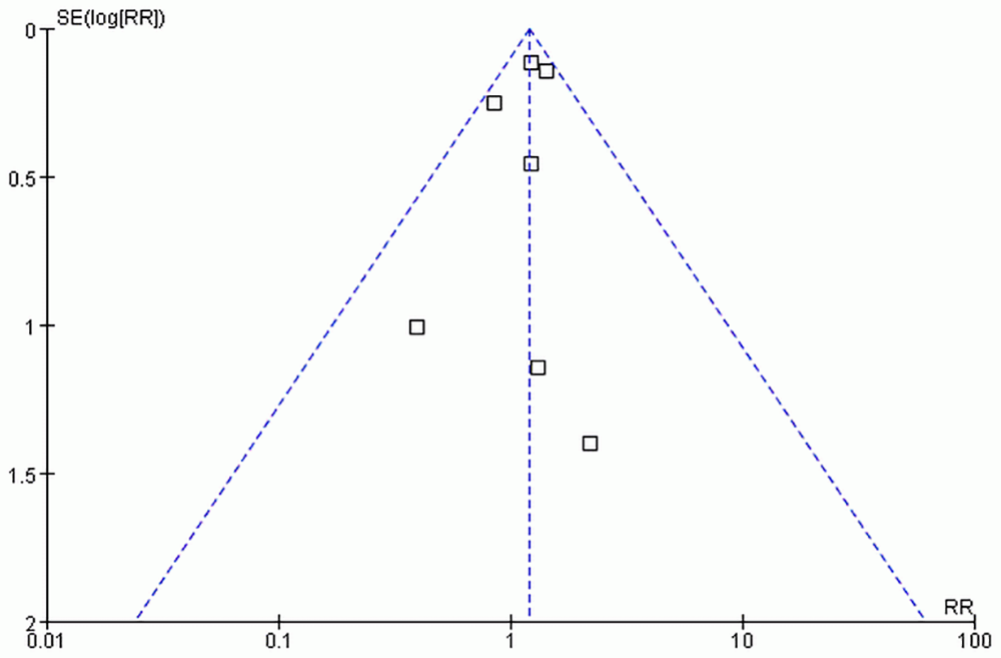
Funnel plot of studies exposure to PTU.

**Fig 6:**
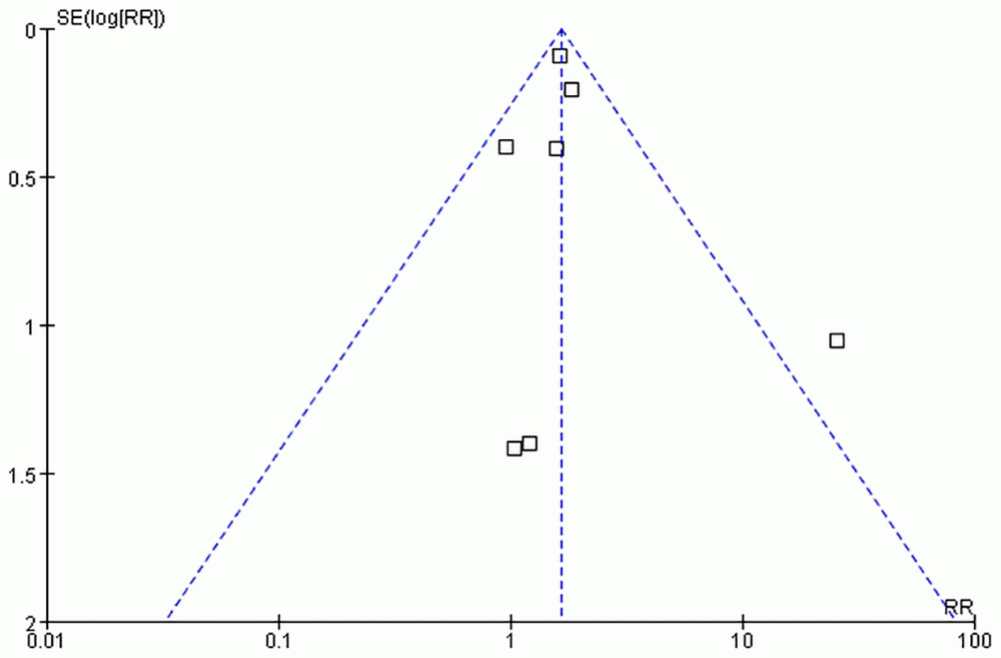
Funnel plot of studies exposure to MMI/CMZ.

The funnel plots of PTU exposure and MMI/CMZ exposure were apparently asymmetric, which provided an evidence of publication bias (Figs 5 and 6). But for PTU & MMI/CMZ exposure, no funnel plot was performed duo to the small number of the included studies.

The fail-safe N numbers (N_*fs*0.05_ = 11 for PTU, N_*fs*0.05_ = 49 for MMI/CMZ, and N_*fs*0.05_ = 7 for PTU&MMI/CMZ) indicated that publication bias was unlikely to affect the pooled result of MMI/CMZ, but likely to affect the pooled results of PTU and PTU&MMI/CMZ.

### Sensitivity analysis

The sensitivity analyses of congenital anomalies were repeated to separately include (1) exposed cohorts involving 30 or more children, (2) unexposed cohorts with no ATD and without hyperthyroidism, and (3) studies with outcomes of major congenital anomalies. Finally, one study [26] was removed from the first sensitivity analysis, since it only involved 28 PTU-exposed children and 12 MMI/CMZ-exposed children. Three studies [18,26,27] were excluded from the second sensitivity analysis. Two studies [2,26] were excluded from the third sensitivity analysis. Table 3 shows the results of pooled RRs and heterogeneity from the sensitivity analyses. The pooled RRs (1.55~1.64) of analysis for MMI/CMZ remained significant and were not heterogeneous (*P* = 0.49~0.98, *I* ^2^ = 0%), which indicated that the results of meta-analysis for MMI/CMZ on congenital anomalies were stable. For PTU, two of the three pooled RRs (1.19~1.27) were significant and not heterogeneous (*P* = 0.58~0.81, *I* ^2^ = 0%), which indicated that the results of meta-analysis for PTU on congenital anomalies were also stable.

**Table 3:** Results of sensitivity analysis.

Included studies	Number of studies	Effect model	RR(95%CI)	*I* ^*2*^	*P*_het_
PTU					
All studies	7	Fixed effect	1.20[1.02, 1.42]	0%	0.60
Sample size not less than 30 in exposed cohorts	6	Fixed effect	1.19[1.02, 1.45]	0%	0.81
Unexposed cohorts with no ATD and without hyperthyroidism	4	Fixed effect	1.27[1.06, 1.51]	0%	0.58
Outcomes with major congenital anomalies	5	Fixed effect	1.18[0.94, 1.49]	10%	0.35
MMI/CMZ					
All studies	7	Fixed model	1.64[1.39, 1.92]	35%	0.16
Sample size not less than 30 in exposed cohorts	6	Fixed effect	1.59[1.35, 1.87]	0%	0.49
Unexposed cohorts with no ATD and without hyperthyroidism	4	Fixed effect	1.55[1.29, 1.85]	0%	0.61
Outcomes with major congenital anomalies	5	Fixed effect	1.64[1.39, 1.94]	0%	0.98

*P*_het_ = *P* value of heterogeneity test

## Discussion

Over the past two decades, many studies have addressed the risk of congenital anomalies in children exposed to ATDs in-utero, but their results are conflicting and inconclusive. There are no comprehensive data regarding the risk of congenital anomalies in children exposed to ATDs in-utero. In this report, we use meta-analysis for the first time to synthesize the pooled RRs of congenital anomalies after exposure to ATDs in-utero. The meta-analysis of 8 cohort studies showed that exposure to either PTU or MMI/CMZ was associated with an increased risk of congenital anomalies. Compared with children not exposed to ATDs in-utero, the risk of congenital anomalies after exposure to PTU, MMI/CMZ, and PTU & MMI/CMZ was increased by 20%, 64%, and 83%, respectively. The findings of our meta-analysis are coincident with a recent systematic review[28], which concludes that both MMI and PTU used in early pregnancy might lead to congenital anomalies in 2%-3% of the exposed children. The systematic review comprehensively describes the effects of ATDs on congenital anomalies reported in previous pertinent studies, but does not report the summary findings in terms of RRs. In the present meta-analysis, the pooled RRs more directly reflect the risks of congenital anomalies in children exposed to ATDs in-utero.

Combining with the results of the sensitivity analyses, we consider the increased risks of congenital anomalies as stable. Previous studies have showed that PTU is considered less teratogenic than MMI/CMZ, and has been recommended as the drug of choice in early pregnancy [7,8,29,30]. Nevertheless, treatment with low dose MMI/CMZ during the second and third trimesters of pregnancy should be considered owing to the association between PTU and liver injury[8,30]. The results in the meta-analysis support the recommended drug treatment for pregnant women with hyperthyroidism, and also can be applied by doctors into pre-conception counseling for reproductive aged women exposed to ATDs. During the time of planning for pregnancy, PTU is the optimal choice for hyperthyroid women in treatment of this disease. Those women who are already taking MMI or CMZ should better switch to PTU once they decide to plan a pregnancy.

It is well-known that the biological half-lives of PTU and MMI in hyperthyroid patients are 2 h, and 6~13 h, respectively. It is generally considered that the drugs can be eliminated from the body after 5 half-lives, and about 3% of the drugs are left in the body[31]. Therefore, the residual effects of MMI and PTU in the body would be eliminated within 3 days and 1 day, respectively. In the meta-analysis, the duration of exposure covers the first trimester of pregnancy in all of the included studies, but it is extended to 6 months before pregnancy in two studies [2,17], and covers the entire pregnancy in three studies [3,18,19]. Some studies confirm that the first trimester of pregnancy is the most important period for the occurrence of most congenital anomalies[12,28]. In the risk estimation of congenital anomalies after exposure to ATDs, we should consider the duration of exposure in the first trimester. The exposure that is extended to 6 months before pregnancy or continues into the second and third trimesters of pregnancy is of little or no importance.

In this study, the meta-analysis method is used to examine the association between the exposure to ATDs in-utero and the occurrence of congenital anomalies, and all the included primary studies are cohort studies. In nearly all case-control studies there are severe problems with recall bias and often large non-response rates. In addition, case-control studies and cohort studies are two different study designs in analytical epidemiology. If the two types of studies were both included in our meta-analysis, the heterogeneity would be intensified. Thus, case-control studies were excluded from the meta-analysis.

As reported, maternal socio-demographic factors (e.g. age, parity, education level, and employment status)[32–34], maternal uncontrolled hyperthyroidism [35,36], maternal other health status[37,38], and genetic factors (e.g. parental family history, and consanguinity)[39–42] are all associated with congenital anomalies. The meta-analysis of 8 cohort studies is complicated by potential differences across studies. Besides the maternal conditions mentioned above, enrollment strategy, measurements of outcomes, and duration of exposure are likely to affect the true effectiveness of ATDs on congenital anomalies. Most of the included studies have considered maternal age, three studies [3,17,18] have adjusted other maternal conditions (parity, education level, pre-gestational disease), but few studies consider maternal thyroid status or the dose of ATDs. Comparisons of the effects on congenital anomalies among different drugs can well address the problem of confounders, after controlling some important factors (e.g. maternal age, severity of hyperthyroidism, and pre-gestational health status) of those participants in each group. The comparison of different ATDs was not conducted in any of the included studies. Hence, such research is necessary in the future, as it would provide more accurate information for risk estimation.

The present meta-analysis has some limitations. First, because the included studies do not provide primary data related to particular congenital anomalies, we are unable to conduct analyses by particular congenital anomalies. As reported, the use of MMI/CMZ is significantly associated with the occurrence of aplasia cutis congenita, choanal atresia, esophageal atresia, and omphalocele [15,16,30], but the rates of these particular congenital anomalies are very low, and most of these studies are case reports. The rates of major congenital anomalies are used in most of the included studies, so the particular congenital anomalies previously reported are not specifically studied in the included studies. Second, the funnel plots are asymmetric, and the fail-safe N numbers are small for PTU and PTU&MMI/CMZ, which indicates the presence of potential publication bias. The publication bias might have unpredictably affected the risk estimation of congenital anomalies. Due to the small number of included studies, we should be careful in making a decision for publication bias according to the results of the funnel plots and fail-safe N. In the meta-analysis, we comprehensively evaluated the pooled results by combining the results of funnel plots, fail-safe N and sensitivity analyses.

In conclusion, this meta-analysis suggests that exposure to ATDs in-utero increases the risk of congenital anomalies. The use of ATDs in pregnancy should be limited when possible. Further research is needed to delineate the exact teratogenic risk for particular congenital anomaly.

## Supporting Information

S1 Checklist PRISMA 2009 Checklist .
(DOC)

S1 Fig PRISMA 2009 Flow Diagram. (DOC)
