# Supplementary material for: Congenital Anomalies in Children Exposed to Antithyroid Drugs In-Utero: A Meta-Analysis of Cohort Studies
Source: PLoS One. 2015 May 14;10(5):e0126610. doi: 10.1371/journal.pone.0126610 (PMC4431808; doi:10.1371/journal.pone.0126610)
Supplement: S1 Fig — (DOC) [file pone.0126610.s002.doc]

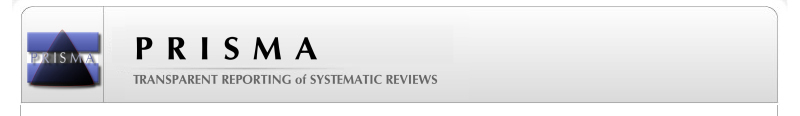
**PRISMA 2009 Flow Diagram**

**Screening**

**Included**

**Eligibility**

**Identification**

Records identified through database searching
(n = 471 )

Additional records identified through other sources
(n = 0)

Records after duplicates removed
(n =310 )

Records screened
(n =310 )

Records excluded
(n = 297 )

Full-text articles assessed for eligibility
(n = 13 )

Full-text articles excluded, with reasons
(n = 5 )

Studies included in qualitative synthesis
(n = 8 )

Studies included in quantitative synthesis (meta-analysis)
(n = 8 )
